# Editorial

**Published:** 1865-07

**Authors:** 


### Editorial.

### EDITORIAL CORRESPONDENCE.

Boston, June 6th, 1865.

Prof. E. Andrews:—*Dear Sir:*—Leaving home on the 18th ult., after a pleasant trip over the Pittsburg, Fort Wayne and Chicago, the Pennsylvania Central, and Northern Central Railroads, we arrived in Baltimore on Saturday morning, where we tarried until the Monday following.

The fields along our route were clothed in their richest green; the forests, in full foliage, were beautified with the white blossoms of the *Cornus Florida*, with here and there a wild rose, a geranium, or a modest violet.

To escape from the din of the city, the unceasing calls of the sick, and never-ending routine of professional life, and enjoy the fresh invigorating air of the country, while the eyes feasted on the hills and valleys, the waving grain, and unbroken forests, was a privilege such as none but the care-worn and toilsome inhabitant of a busy city knows how to appreciate.

After resting over the Sabbath in Baltimore, we proceeded, on Monday morning, to Washington, where we expected to spend two or three days. But such was the throng, in anticipation of the great military reviews to take place on Tuesday and Wednesday, that we could not find a lodging in any kind of a hotel in the city. About noon, however, we obtained room in a private boarding-house, and hastened to the office of the Surgeon-General, for the purpose of gaining admission to the Army Medical Museum. We (myself and son) were very kindly received by Surgeon-General BARNES, who sent one of his assistants, immediately, to show us everything of interest in the Museum.

We found the collection neatly and conveniently arranged, though in a building too small for the number of specimens that are still rapidly increasing. I was particularly interested in a series of wet pathological specimens, showing the changes in the mucous membrane of the intestines, effected during the progress of camp diarrhœa, dysentery, and typhoid fever. The specimens exhibit traces of disease in almost every stage of progress, from simple redness or slight inflammatory action, to that of extensive ulceration and gangrene. The specimens of gunshot injuries are very numerous, and those of the larger joints intensely interesting. The results of exsections are presented in considerable numbers, and, so far as relates to the knee and other hinge joints, they certainly do not encourage this branch of conservative surgery. With the shoulder and hip it is different. In the collection, there are several skulls, showing only a very slight graze or contusion of a ball upon the exterior surface, while the inner table was fully and *comminutely* fractured. One of the most interesting and instructive features of this museum, is the photographic plates and illustrations, both life-size and microscopic. The specimens in the museum are all neatly mounted, labelled, and their histories, as far as possible, carefully preserved. We should think the work of the whole establishment was in good and faithful hands, and the profession have reason to expect much benefit from it in the future.

On Tuesday morning, all public places were closed, Pennsylvania Avenue thoroughly washed by the fire-engines, to prevent dust, and the assembled thousands, from every part of the country, of all ages, colors, and sexes, began to gather in front of the Presidential mansion, and the streets leading to the Avenue, to witness the grand review of the old Army of the Potomac. Avoiding the thickest of the crowd, we (myself, wife and son) took our stand on Pennsylvania Avenue, near Capitol Hill, where, precisely at the appointed hour, we saw Major-General MEADE and full staff, as they swept around the Hill into the Avenue, and up towards the White House, followed by 75,000 cavalry, artillery, and infantry, with their martial music and banners, receiving the welcome shouts of the multitudes, and frequent presents of flowery wreaths from fair hands. The day was clear, the air pure and bracing, and we sat gazing with mingled emotions of awe and gratitude upon brigade after brigade of those war-worn and faithful defenders of their country, until the last man of that mighty column had passed, when, on noting the time, we found that it had been full five and a-half hours. The remainder of the day was spent in the Smithsonian Institute and around the public grounds.

The following morning, we again took up our position on Pennsylvania Avenue, to see the grand army made up of our Western boys make their hard-earned triumphal entry into the capital of the nation. Exactly at the moment specified in the general orders, the well-known form of Major-General W. T. SHERMAN was seen entering the Avenue at the foot of Capitol Hill, accompanied by his staff, all mounted on superb horses, closely followed by the body-guard and full band of music. Then came the dense column of veterans, twenty abreast, with bayonets glittering in the sun, their tattered colors flying, while the shouts of the hundreds of thousands of spectators were echoed and reëchoed from Capitol Hill to the White House. The enthusiasm and delight of the people, exhibited on Tuesday, was repeated and immensely increased as the noble army that had literally traversed the continent victoriously from Cairo to the Gulf, and from the Gulf to the Atlantic coast of the Carolinas, passed with firm and steady step, to pay their respects to the President and Lieutenant-General of the nation.

The grand and glorious spectacle of those two days has scarcely been equalled in the military history of the world.

To avoid the crowd which would certainly render all the evening railroad trains out of Washington uncomfortable, we hastened from the sublime spectacle in Pennsylvania Avenue to the 11 o'clock A.M. train for Philadelphia, where we arrived safely in the evening. While in Philadelphia, I visited the Meuter Museum, where I found an excellent building, sufficient to accommodate a good pathological and anatomical museum, a good library, and a pleasant lecture-room. It is under the management of the College of Physicians and Surgeons of Philadelphia. In the library-rooms there is a pretty extensive and valuable collection of medical books, some of which are very ancient and rare. The articles in the museum, however, disappointed me both in number and quality. After visiting Laurel Hill, Girard College, and spending half-a-day profitably in the Museum or Hall of the Academy of Natural Sciences, in Philadelphia, we took our leave and started for New York, where we arrived on Saturday evening. Here, wishing chiefly to invigorate the physical system, and gratify the social feelings, we avoided scientific and professional matters as much as possible, and spent a week in trips to Staten Island, Coney Island, Central Park, &c., enlivened with the society of old friends.

As a meeting of the Academy of Medicine took place one evening during the week, we accepted an invitation to accompany one of its members. The subject for discussion was Pyæmia, on which an interesting paper had been read at a previous meeting, by Dr. Krackowitzer. A goodly number of members were in attendance, but they seemed to enter upon the discussion of the important subject before them with much reluctance. After considerable delay, the discussion was finally commenced by Dr. E. R. Peaselee, and was continued by Drs. F. H. Hamilton and B. F. Barker. It was conceded by all the speakers, as well as by the paper of Dr. Krackowitzer, that the symptoms usually attributed to pyæmia are not produced by the absorption of *healthy* pus, but by some septic poison which may be developed by decomposition of pus, or, sometimes, without the presence of any perceptible suppurative action at all. Hence the name pyæmia was regarded as objectionable.

On Friday afternoon, we entered a steamer, bound for Providence. After a delightful trip through the East River, in full view of the islands containing the charitable and criminal institutions of the great commercial metropolis of the country, we entered the broader part of Long Island Sound, and the darkness of night shut the surrounding objects from view. On awakening from a good night's sleep, we were rapidly nearing Providence, where we passed directly into the rail-car, and arrived in Boston in time for an early breakfast. Here we find every preparation for a good meeting of the American Medical Association. And, as it is to be called to order at 10 o'clock A.M., I must close this letter. Yours truly, N. S. D.

---

AMERICAN MEDICAL ASSOCIATION.—The above letter, addressed to my colleague, Dr. ANDREWS, was intended to help fill up the editorial space in the June number of the EXAMINER. But my colleague had kindly filled the space, and had the whole number in type, before the letter was received.

The recent meeting of the Association at Boston, was one of the largest and most profitable that has ever been held. Over six hundred members were present. For earnestness in devotion to appropriate business; cordial friendship among members; and true social enjoyment, it may be said to have excelled all the previous meetings. The arrangements made by the local committee in Boston, were very judicious and liberal; the several sections were promptly organized, and commenced their work on the afternoon of the first day; and though the meetings of the Association were protracted to 1 o'clock P.M., of the fourth day, yet from the first to the last hour of the time, promptness, regularity, close attention, and perfect cordiality, characterized every step. The citizens and city authorities of Boston, were most liberal in their attentions to the Association. The local profession of Boston, the municipal authorities, and the Governor of the Commonwealth, are alike entitled to the thanks of the whole profession, for their generous hospitality and judicious arrangements.—If there were any in the profession who imagined that the American Medical Association had lost its original vitality, and was doomed to a rapid decline, especially if *they* withheld *their* presence and influence, they cannot fail to see in the number, earnestness, and cordiality of the recent meeting in Boston, very clear evidence of their mistake. On the contrary, we think its career of usefulness and real activity has only just begun.

---

SURGEON-GENERAL BARNES AND HOMŒOPATHY.—In a recent number of the *Medical and Surgical Reporter*, of Philadelphia, it was stated that at the recent meeting of the American Medical Association, resolutions were introduced censuring Surgeon-General Barnes, for having consulted with a homœopathic practitioner in the case of Secretary Seward, in Washington; and that after proper explanations they were withdrawn.

This is a mistake. No resolutions were introduced on the subject, and, of course, none were withdrawn. During the progress of the late meeting at Boston, a letter was received from Dr. W. H. Mussey, of Cincinnati, one of the Vice-Presidents of the Association, explaining that his absence from the meeting was on account of sickness in his family; and, incidentally, alluding to the alleged fact that the Surgeon-General of the U.S. A. had been in consultation with a homœopath in such a manner as to deserve severe censure. This letter, in accordance with the usual custom, was read to the meeting. That part of it relating to the Surgeon-General caused a representative from the Medical Department of the Army to rise and fully and *authoratatively* deny the truth of the allegations against that officer. This being entirely satisfactory to the Association, the subject was then dropped, as no formal question had been presented for the action of the meeting.

---

THE NEW DISPENSATORY.—Physicians should observe that in the new Dispensatory the strength of many of the preparations is changed, a fact which needs to be carefully noticed in prescribing. In many respects the new preparations are superior to the old.

Mortality of Chicago for May, 1865.—Furnished by the Assistant Health Officer, Matt. P. Riordan.

DISEASES.

| | | | |
|---|---|---|---|
| Accident, | 5 | Bilious Fever, | 5 |
| Apoplexy, | 1 | Inflammation of Brain, | 7 |
| Burned, | 1 | Inflammation of Stomach, | 1 |
| Catarrh, | 1 | Inflammation of Bowels, | 4 |
| Congestion of Brain, | 4 | Inflammation of Lungs, | 4 |
| Congestion of Lungs, | 2 | Inflammation of Bladder, | 1 |
| Consumption, | 32 | Intemperance, | 2 |
| Croup, | 17 | Killed, | 2 |
| Cramps, | 13 | Lock Jaw, | 1 |
| Convulsions, | 6 | Measles, | 2 |
| Childbirth, | 6 | Marasmus, | 1 |
| Cancer of Stomach, | 2 | Old Age, | 5 |
| Cerebro-Spinal Meningitis, | 4 | Poisoned, | 1 |
| Congestive Chills, | 2 | Paralysis, | 2 |
| Dysentery, | 2 | Pneumonia, | 2 |
| Diphtheria, | 2 | Rheumatism, | 2 |
| Disease of the Heart, | 3 | Small-Pox, | 5 |
| Dropsy, | 6 | Stillborn, | 8 |
| Diarrhœa, | 9 | Strangulated Hernia, | 1 |
| Erysipelas, | 3 | Teething, | 6 |
| Nervous Fever, | 2 | Unknown, | 6 |
| Scarlet Fever, | 8 | Water on Brain, | 5 |
| Typhoid Fever, | 6 | Worms, | 3 |
| Brain Fever, | 6 | | |
| Spotted Fever, | 1 | Total, | 229 |
| Puerpural Fever, | 1 | | |

Ages of the Deceased. — Under 5 years, 91; over 5 and under 10 years, 19; over 10 and under 20, 12; over 20 and under 30, 34; over 30 and under 40, 22; over 40 and under 50, 12; over 50 and under 60, 14; over 60 and under 70, 7; over 70 and under 80, 3; over 80 and under 90, 1; over 90 and under 100, 3; stillborn, 8; unknown, 3. Total, 229.

NATIVITIES.

| | | | | | |
|---|---|---|---|---|---|
| Chicago, | 107 | Scotland, | 1 | Illinois, | 3 |
| Canada, | 4 | Sweden, | 1 | Other States, | 41 |
| Colored, | 1 | France, | 1 | Unknown, | 2 |
| Belgium, | 0 | Germany, | 24 | Wales, | 1 |
| England, | 6 | Holland, | 1 | | |
| Ireland, | 35 | Norway, | 1 | Total, | 229 |

DIVISIONS OF THE CITY.

North, 48 | South, 88 | West, 93 | Total, 229

SMALL-POX.

North, 1 | South, 2 | West, 2 | Total, 5

Total number of deaths in May, 1864, 309 | Small-Pox, 30
Total number of deaths in April, 1865, 256 | Small-Pox, 4

Electrical Anæsthesia.—In a short article in the Lombardy *Gazette Medica*, March 13, an account is given of a demonstration made by Dr. Rodolfi, at the Brescia Hospital, in the presence of a large number of medical practitioners, of the power of the electrical current to induce local anæsthesia. This is complete enough to admit of the execution of painless surgical operations, although it does not appear that any such have as yet been performed; and it is remarkable in its extreme duration, three days been spoken of as a common period. It seems that women (especially when nervous or hysterical) are more susceptible of its action than men, and it fails to produce any anæsthetic action in about 6 per cent. of the individuals submitted to it. In a case of a woman acted upon before the witnesses, anæsthesia of the hands began to be induced ten minutes after the application of a continuous current from a Bunsen's pile with six elements; and another woman was exhibited in whom complete anæsthesia had affected the whole surface above the diaphragm for three days. An hysterical subject manifested general anæsthesia, with paresis of movement of the limbs, and submitted to have her tongue traversed with a needle. In her case the anæsthesia continued from ten to fifteen days, gradually diminishing. The intellectual faculties continue in these cases quite undisturbed. In one of the cases experimented upon anæsthesia could not be induced, which Dr. Rodolfi attributes to the aversion the patient felt to the presence of so large a number of spectators.—*Medical Times and Gazette.*

---

A SIMPLE OPHTHALMOSCOPE.—[*From the British Medical Journal.*]—SIR:—I find that if a convex lens of about two inches focus be placed in close apposition with a concave one of about nine inches focus, and this combination be held before the patient's eye, at the distance the object-lens of an ordinary ophthalmoscope usually is, it forms an ophthalmoscope, uniting in itself the reflecting and refracting elements of that instrument. For, whilst the light from a flame is reflected by two surfaces (the outer concave surface of the concave lens and the internal concave surface of the convex one) into the patient's eye, it is also, on its emergence therefrom, refracted by the effective convex element of the combination, so as to form the usual indirect image of the fundus oculi at the focal length. With such a rough combination, I have been able to obtain a distinct image of the optic nerve, retinal vessels, etc.; and I may hence not unreasonably hope a properly constructed meniscus will in itself fulfil the conditions of the mirror and object-lens of an ordinary ophthalmoscope.

I am, etc., J. Z. LAURENCE.

30 Devonshire Street, Portland Place, May 3d, 1864.

HEADQUARTERS ARMY OF THE TENNESSEE,
MEDICAL INSPECTOR'S OFFICE,
*Louisville, Kentucky, June 28th*, 1865.

DOCTOR:—Will you please give notice, through the EXAMINER, that fifteen or twenty physicians are wanted in this army, to serve with the troops encamped about the city. They can be employed under contract, for at least three months, by applying in person at the Headquarters of the Army of the Tennessee, in Louisville, Ky.

Surgeons and assistant-surgeons, who have served in the field during the war, are anxious to return to their homes. Many have already left the service, making the demand for medical officers very great. Yours respectfully,

JNO. M. WOODWORTH,

*Med. Insp. Army of the Tennessee.*

PROF. N. S. DAVIS,

*Chicago, Ill.*

P.S. The monthly compensation for medical officers under contract in the field, is $113,83 per month, and furnished with horse and equipments. The medical officer furnishes his own instruments. WOODWORTH.

---

HEALTH OF THE PACIFIC COAST.—As far as our knowledge extends, the health of this coast, for the last six months, has been uncommonly good. Malarious fevers, generally of a mild type, have prevailed endemically, to a slight extent, and diphtheria has dropt like a deadly meteor on a few localities, and then vanished. The season was very wet in the beginning, and very dry subsequently, but no morbific influence appears to have attended the climatic irregularity.

---

TREATMENT OF HYDROCELE.—Dr. William Jollie (Gateshead-on-Tyne) recommends the following plan, which he has followed for some years, and invariably with success, even after failure with port wine, &c.:—

"I tap the hydrocele by trocar and canula in the usual way, draw off the fluid, and then introduce through the canula into the cavity of the tunica vaginalis a common surgeon's probe, which has been previously coated, for an inch of its length, with nitrate of silver. I prepare the probe by heating the extremity to a dull red heat in the flame of a gaslight, and placing it in a little finely-powdered nitrate of silver, and then again subjecting the probe to the heat, so as to form a smooth coating to the instrument. If your correspondent, and other surgeons, will make use of this method, they will, I have no doubt, quickly, effectually, and cheaply relieve their patients of a troublesome complaint."

---

Will Oleum Tanaceti Cause Abortion.—This question was discussed at a late meeting of the Buffalo Medical Association, as reported in the *Buffalo Medical and Surgical Journal.* A woman who had used it died in consequence, and on inspection extensive peritonitis was exhibited, with purulent effusion. Other fatal cases were reported, and the general conclusion was that the agent had no specific effect on the uterus, and often destroyed life without producing abortion. The loss of the fœtus, when that takes place, was ascribed to the violence done to the organs generally by the poisonous drug.

